# ASAP - A Webserver for Immunoglobulin-Sequencing Analysis Pipeline

**DOI:** 10.3389/fimmu.2018.01686

**Published:** 2018-07-30

**Authors:** Oren Avram, Anna Vaisman-Mentesh, Dror Yehezkel, Haim Ashkenazy, Tal Pupko, Yariv Wine

**Affiliations:** George S. Wise Faculty of Life Sciences, School of Molecular Cell Biology and Biotechnology, Tel Aviv University, Ramat Aviv, Israel

**Keywords:** high throughput sequencing, antibodies, B cell receptor, next generation sequencing, Ig-Seq, AIRR-Seq, antibody repertoire analysis, immune repertoire

## Abstract

Reproducible and robust data on antibody repertoires are invaluable for basic and applied immunology. Next-generation sequencing (NGS) of antibody variable regions has emerged as a powerful tool in systems immunology, providing quantitative molecular information on antibody polyclonal composition. However, major computational challenges exist when analyzing antibody sequences, from error handling to hypermutation profiles and clonal expansion analyses. In this work, we developed the ASAP (A webserver for Immunoglobulin-Seq Analysis Pipeline) webserver (https://asap.tau.ac.il). The input to ASAP is a paired-end sequence dataset from one or more replicates, with or without unique molecular identifiers. These datasets can be derived from NGS of human or murine antibody variable regions. ASAP first filters and annotates the sequence reads using public or user-provided germline sequence information. The ASAP webserver next performs various calculations, including somatic hypermutation level, CDR3 lengths, V(D)J family assignments, and V(D)J combination distribution. These analyses are repeated for each replicate. ASAP provides additional information by analyzing the commonalities and differences between the repeats (“joint” analysis). For example, ASAP examines the shared variable regions and their frequency in each replicate to determine which sequences are less likely to be a result of a sample preparation derived and/or sequencing errors. Moreover, ASAP clusters the data to clones and reports the identity and prevalence of top ranking clones (clonal expansion analysis). ASAP further provides the distribution of synonymous and non-synonymous mutations within the V genes somatic hypermutations. Finally, ASAP provides means to process the data for proteomic analysis of serum/secreted antibodies by generating a variable region database for liquid chromatography high resolution tandem mass spectrometry (LC-MS/MS) interpretation. ASAP is user-friendly, free, and open to all users, with no login requirement. ASAP is applicable for researchers interested in basic questions related to B cell development and differentiation, as well as applied researchers who are interested in vaccine development and monoclonal antibody engineering. By virtue of its user-friendliness, ASAP opens the antibody analysis field to non-expert users who seek to boost their research with immune repertoire analysis.

## Introduction

**Figure 1:**
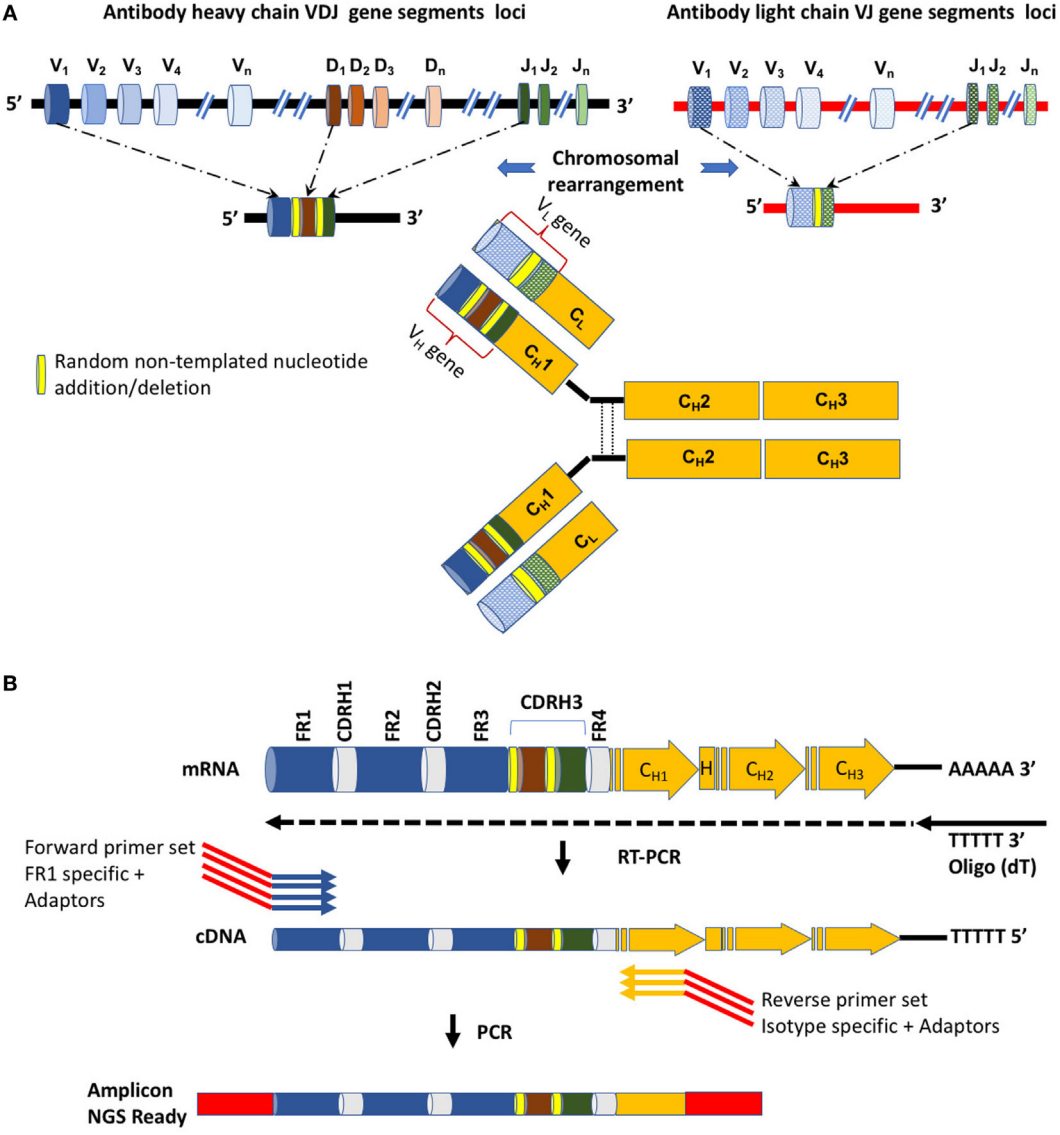
The diversity of antibody sequences and structures and molecular methodologies for next-generation sequencing. **(A)** Antibodies are comprised of two identical heavy chains and two identical light chains, each encoded on a different chromosome, both in human and in mouse. Diversity is achieved by chromosomal rearrangement, where different V, D, and J (V and J) genes are combined to construct the variable region of the heavy (light) chain of the antibody. In yellow are random nucleotides introduced during the chromosomal rearrangement process. **(B)** A detailed view of the variable region. Shown are the forward and reverse primers used for amplification. Several alternative primers, both forward and reverse, are used in order to capture the diversity of the variable region and its associated isotypes. The forward primers anneal to the framework 1 (FR1) region. Red regions within the primers represent adaptor sequences.

The power of the adaptive immune system relies on its ability to generate an exceptional diversity in the genes encoding the variable region of B cell receptors and their secreted form, the antibodies. This diversity of antibodies is achieved by several unique molecular mechanisms, including chromosomal V(D)J rearrangement during B cell maturation in the bone marrow, N-P addition/deletion in the ligated V(D)J genes and somatic hypermutations (SHM) following antigen stimuli in the peripheral lymph nodes (1) (Figure 1A).

Antibodies can reach an enormous theoretical diversity of 10^13^–10^18^. However, the actual diversity is more restricted and was estimated to reach 10^11^–10^12^ in humans (2). Due to labor and cost considerations, as well as the lack of suitable high-throughput technologies, analysis of such complex repertoires using traditional Sanger sequencing was impractical for many years, resulting in major knowledge gaps regarding antibodies molecular composition. This precluded the ability to address many fundamental immunological questions related to the development of the immune response in health, disease, and following vaccination.

The introduction of next-generation sequencing (NGS) platforms has significantly advanced research in many scientific fields and opened new avenues in genomics and transcriptomics research. For immunoglobulin sequencing (Ig-Seq), which is also termed adaptive immune receptor repertoire sequencing (AIRR-Seq), NGS provided the means to underline quantitative measures of the immune response in an unprecedented throughput and since 2009 (3), the number of studies that applied NGS to analyze immune repertoires has increased substantially.

AIRR-Seq is based on targeted sequencing of genomic DNA or mRNA and in principle focuses on recording the diversity of the variable region (which includes the V(D)J genes) encoding the heavy (V_H_) and/or light (V_L_) chains of antibodies (Figure 1B). The variable region encodes the most diversified sites of the antibodies as they are the product of chromosomal rearrangements and SHMs and comprise three complementary determining regions (CDR1-3) in each antibody chain (Figure 1B). Due to the recombination and non-templated diversification mechanisms that generate the CDR3 of the heavy chain (CDR-H3), it is considered the most diverse determinant in terms of length and sequence of AIRR. CDR-H3 is thus pivotal for antibody specificity, although it was recently suggested that CDR-H3 is necessary, albeit insufficient, for specific antibody binding (4).

Accumulating AIRR-Seq data provide invaluable insights regarding the nature of the immune response in health and disease. These data were shown to be important for isolation and expression of antigen-specific monoclonal antibodies (5, 6), sequencing and cloning antibodies from single cells (7, 8), and proteomic analyses of secreted antibodies (9–11). These sequencing data can further facilitate the elucidation of the properties of antigen-specific antibodies that mediate protection against infectious diseases, are induced following vaccination, and generated in cancer and autoimmune diseases.

While AIRR-Seq is a powerful tool for immune repertoire analysis, errors accumulated during the experimental procedure (e.g., PCR and sequencing errors) make it extremely difficult to confidently/reliably determine the qualitative and quantitative measurements of the immune repertoire and establish an error-free antibody variable region sequence database. High confidence antibody variable region archives are particularly important when AIRR-Seq is combined with the utilization of serum antibodies proteomics (12–16), as these archives define the search space to interpret the proteomic spectra.

To overcome these challenges, experimental and computational strategies can be employed to reduce error-derived “noise” (17, 18). One such strategy utilizes replicate samples (either technical or biological) (19, 20). The main advantage of this approach is that it does not require complex experimental protocols that may prevent researchers from exploring the potential usage of AIRR-Seq in their research. Noteworthy, while great effort is invested in the development AIRR-Seq analysis tools, there is still no consensus on standard operating procedures for data processing and deposition. To address these issues, the AIRR Community was established in 2014 (http://airr.irmacs.sfu.ca/home) (21, 22).

Here, we report ASAP (A webserver for Ig-Seq Analysis Pipeline), a webserver for the analysis of AIRR-Seq data from several replicates, that is user-friendly, simple, free, and open to all users. ASAP is easily accessible to researchers who are interested to address basic questions related to B-cell development and differentiation in health and disease, as well as to researchers interested in applicable vaccine development and monoclonal antibody engineering. ASAP provides several unique features that are absent from other published webservers dedicated for AIRR-Seq data processing, analysis, and visulation (Table 1). ASAP and its associated source code are freely available at https://asap.tau.ac.il and https://github.com/orenavram/ASAP, respectively.

**Table 1:** Summary of the analyses supported in ASAP compared to other related webservers.

Feature	ASAP	BRepertoire	ARGalaxy	Vidjil	VDJviz	VDJServer
Paired-end alignment	+	−	+	+	−	+

VDJ annotation	+	−	+	+	−	+

Framework and CDR3 annotation	+	−	+	+	−	+

Filtering	+	−	+	+	−	+

Data trimming	+	−	+	+	−	+

Unique molecular identifier clustering	+	−	−	−	−	+

Inclusion of novel germline alleles	+	−	−	+	−	−

CDR3 length distribution	+	+	+	+	+	+

Somatic hypermutations level	+	−	+	−	−	+

Synonymous/non-synonymous mutations	+	+	+	−	−	+

Isotype distribution	+	−	+	−	−	−

Joint analysis for replicates	+	−	−	−	−	+

Clonal analysis	+	+	+	+	+	+

V(D)J usage	+	+	+	+	+	+

V-D-J combination analysis	+	+	+	+	+	+

Clone ranking detailed analysis	+	–	−	−	+	+

Integrative database of all unique variable region sequences including sequence metadata	+	–	−	−	−	−

MS ready database for proteomics analysis of secreted antibodies	+	–	−	−	−	−

Amino acid level sequences linked to nucleotide level	+	–	−	−	−	−

Single push-button for all analysis steps	+	–	+	−	−	−

## Results

The webserver ASAP allows analysis of the complete B cell receptor repertoire based on NGS replicates of antibody variable region sequencing experiments. Implemented in Python 3, ASAP is simple, user-friendly, and freely available for all users.

**Figure 2:**
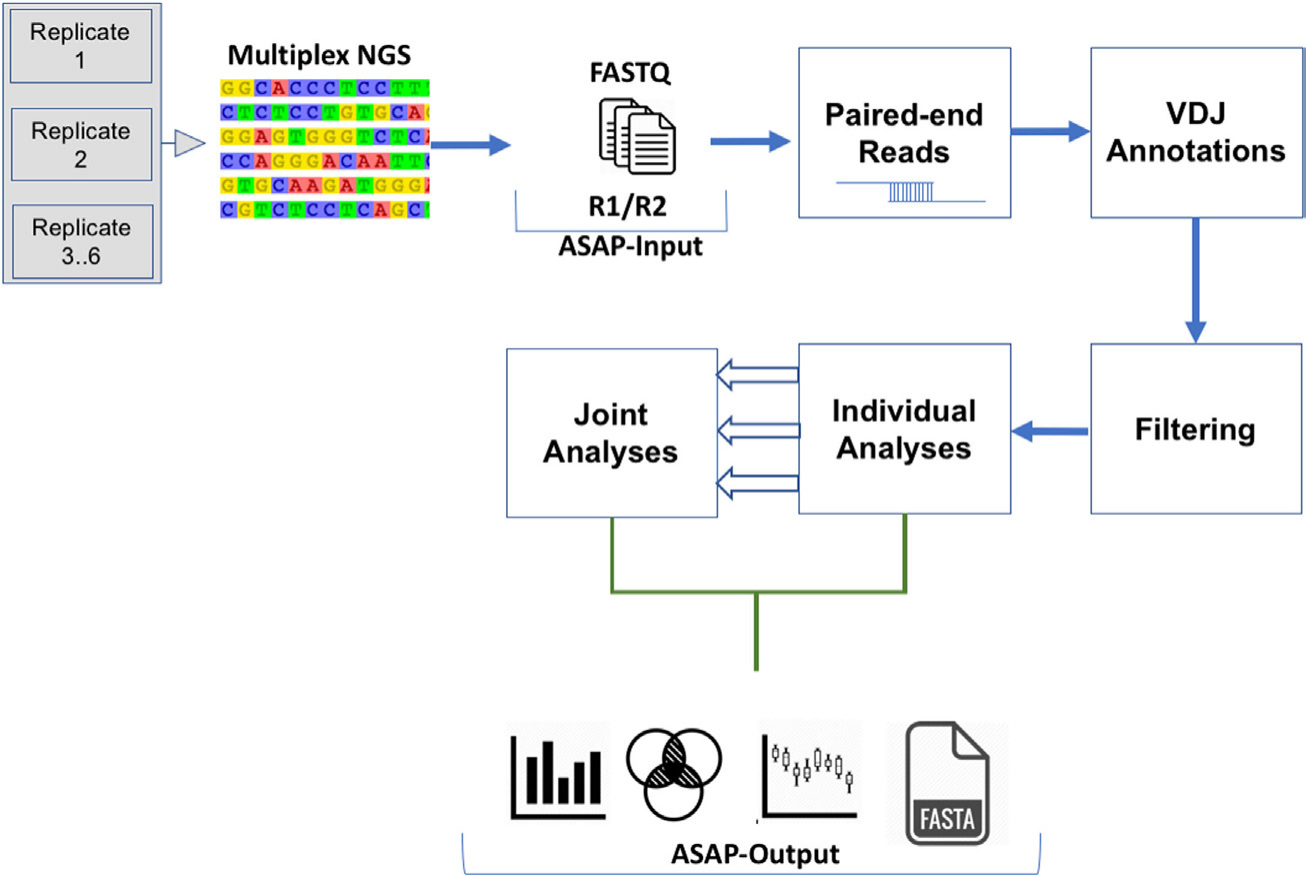
Schematic flowchart for the analysis of each next-generation sequencing replicate (individual) as well as the analyses of the entire set of replicates (joint).

The simplicity of the webserver allows researchers of diversified expertise levels to submit up to six replicates, given that the replicates use different barcodes. ASAP consists of two major parts: the individual part, in which each replicate is analyzed separately, and a joint part, in which the commonalities and differences among the replicates are analyzed. A complete overview of the ASAP workflow and output information is shown in Figure 2. A detailed description of all types of analyses provided by ASAP can be found on the webserver’s Gallery section.

To exemplify the advantages of ASAP, we present here a demonstration of the entire webserver workflow by analyzing previously published antibody sequence data (23). These data were obtained from murine pools of plasmablasts and plasma cells in technical triplicates, i.e., three samples prepared from the same starting cDNA pool. All samples were sequenced using Illumina MiSeq platform, 2 × 250 bp paired-end reads (European nucleotide archive study accession: PRJEB4643).

### Individual Processing

The input to ASAP is two FASTQ files for each replicate (paired end files). These files are initially processed by the ASAP webserver using the MiXCR software (24), which provides full V(D)J assignment, frameworks, and CDR3 annotations.

Alignment files that were generated by MiXCR are further processed. Aligned sequences are filtered out if at least one of the following conditions is met: (1) sequence contains a stop codon in the variable region ORF; (2) the two paired-end reads do not overlap; (3) the obtained sequences are shorter than a specified threshold (default set to be 300 nucleotides); (4) read quality is lower than a specified threshold (default set to be 20). A file describing the number of sequences filtered due to each criterion is provided. In case the reads are associated with a unique molecular identifier (UMI), reads with the same UMI are collapsed to a single sequence and errors are corrected based on the consensus sequence (25). For UMI analyses, the user has to provide the UMI pattern according to the IUPAC nucleotide code. Notably, UMI are handled in cases where the UMI is found only on the forward read, only on the reverse read, or both, as described in Ref. (19).

Each chain type has a different output section as follows. The first output of the processed data is an annotation file (e.g., “IGH_aa_sequence_annotations,” for the IGH chain). In this file, each row contains the following information regarding unique amino acid sequences (identical sequences are grouped to a single line): [1] chain type (V_H_, V_κ_?, or V_λ_); [2] antibody isotype associated with the V_H_ sequences (e.g., for human sequences: IgM, IgD, IgG, IgA1, IgA2, or IgE); [3] the trimmed nucleotide read (without the adapter sequence); [4] the corresponding amino acid sequence; [5] amino acid sequence of the CDR3 region; [6] V family subgroup; [7] D family subgroup; [8] J family subgroup; [9] the number of reads for this amino acid sequence (counts).

**Figure 3:**
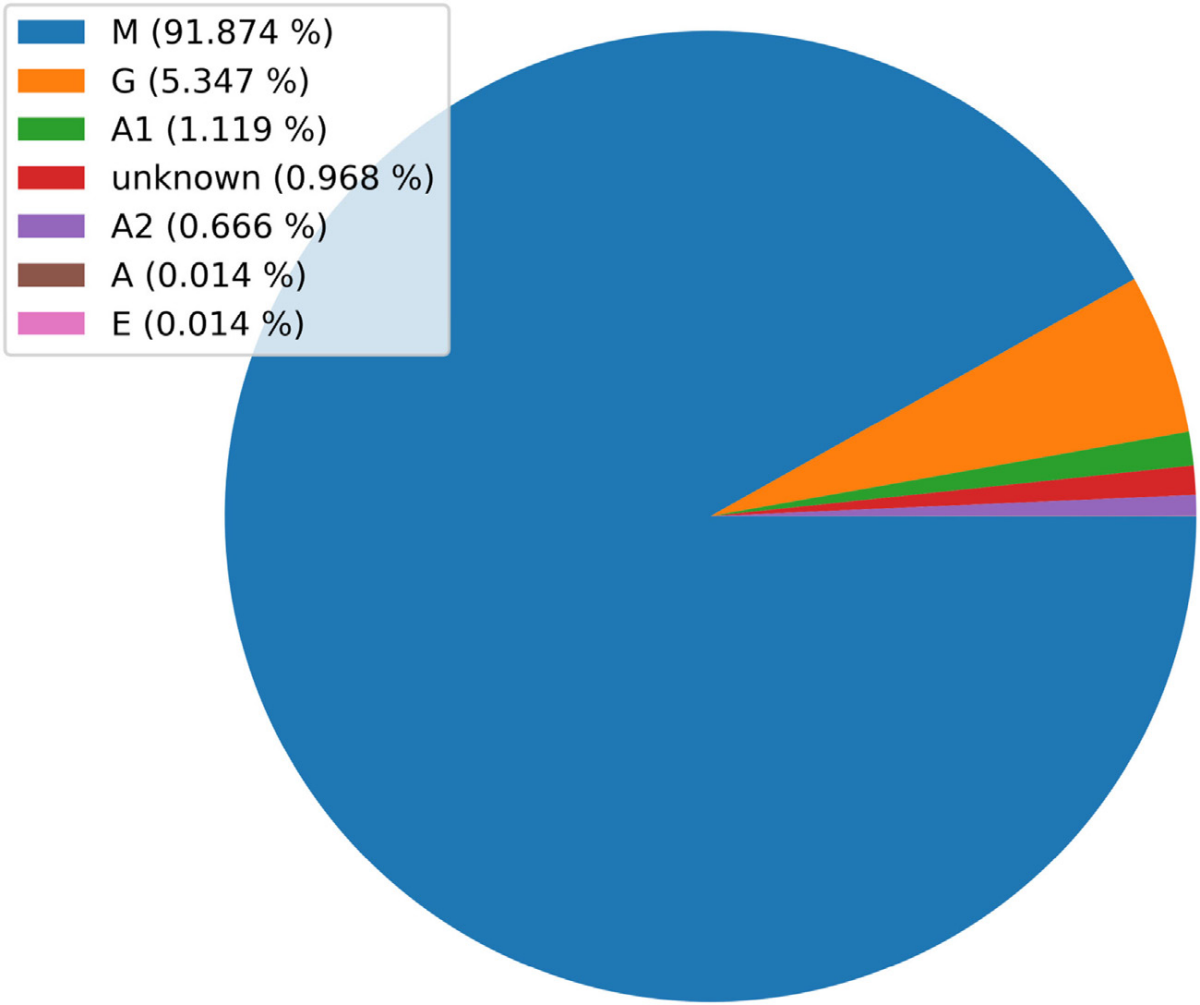
A pie chart showing the distribution of isotypes in a specific next-generation sequencing (NGS) replicate. Note, this chart was generated using unpublished human NGS data.

The isotype assignment is computed by string matching to peptides defining each isotype. These peptides correspond to the N terminus of the antibody C_H_1 region (Figure 1A) and were derived from IMGT (26) (Table 2). Isotypes are assigned by searching for an exact match between a substring of the translated read and these peptides. Specifically, the peptides are searched against the C terminal region to the Framework-4. This region is defined by the string VTVSS in human and by the strings VTVSS, LTVSS, and VTVSA in mouse. In case no match is found, the server searches for the closest match. In case the difference between the closest match is more than a single amino acid mismatch, the isotype is classified as “unknown.” In addition, for certain human samples, the server is unable to distinguish between the A1 and A2 isotypes, e.g., when the relevant peptide motif information used for this classification is ambiguous (the peptide motif ends with CSTQP for A1 and DSTPQ for A2; Table 2). In this case, the isotype is defined as “IgA.” This isotype information is included in the annotation file described above. The frequencies of each isotype are graphically presented as a pie chart (Figure 3). Of note, the ability to detect the various isotypes depends on the primers used within the experimental setup.

**Table 2:** The sequence fingerprint characteristic of each isotype in human and mouse.

Isotype	Human	Mouse
M	GSASAPT	ESQSFP
D	APTKAP	GDKKEP
G	ASTKGPS	AKTT[A/P]P
A	ASPTSP	ESARNP
A1	ASPTSPKVFPLSLCSTQP	–
A2	ASPTSPKVFPLSLDSTPQ	–
E	ASTQSP	ASIRNP

**Figure 4:**
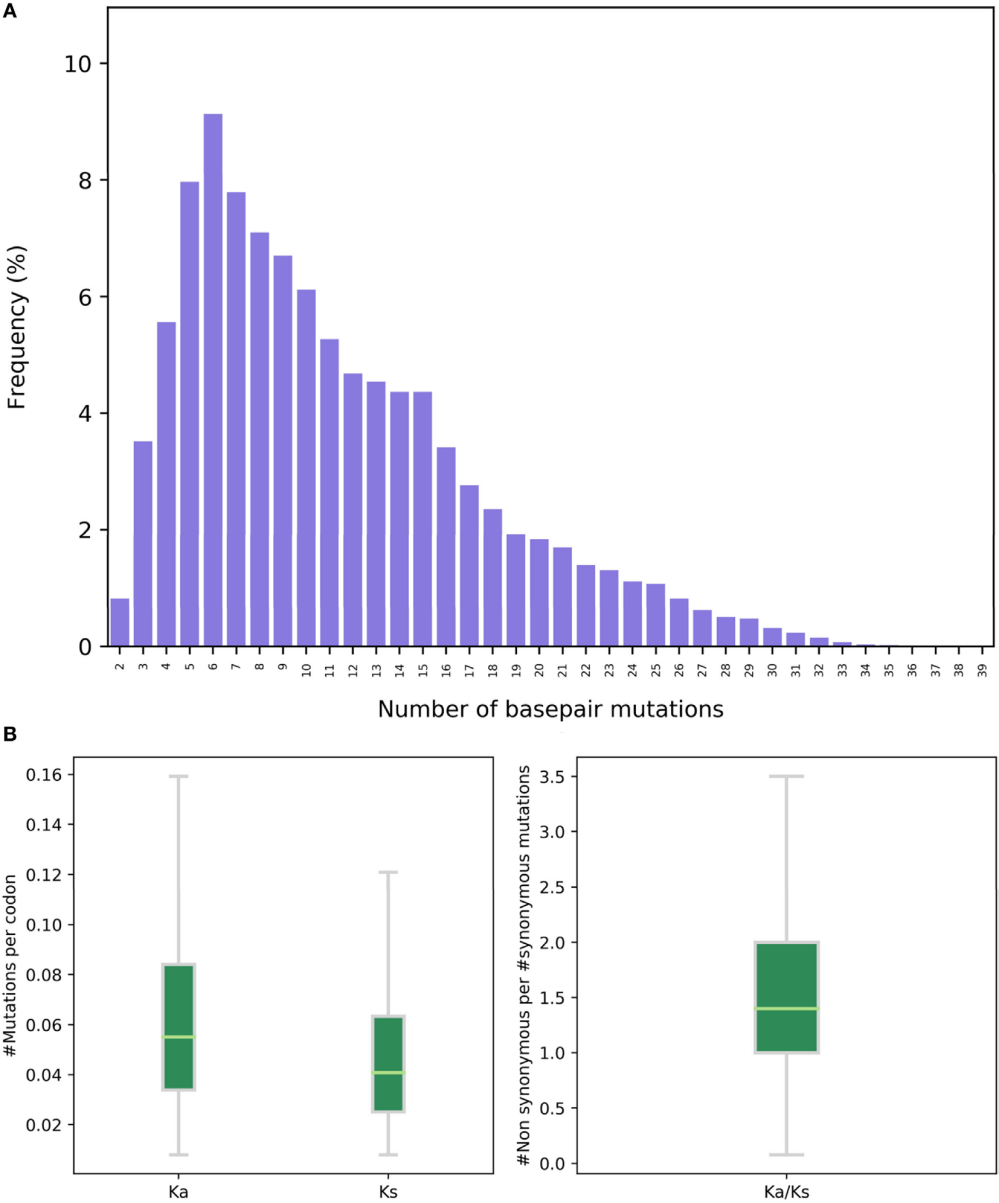
Somatic hypermutation analysis. **(A)** A histogram showing the frequency of the number of base pair mutations in a next-generation sequencing replicate. The *X* axis represents the number of mutations (both synonymous and non-synonymous) defined by comparison to the germline genes. **(B)** The number of non-synonymous (Ka) and synonymous (Ks) mutations and their ratios (Ka/Ks), based on comparison to the germline genes. The *Y* axis is the number of mutations per codon. Each dot represents a unique variable region nucleotide sequence.

Next, ASAP provides information regarding SHM. For each DNA read, the number of mutations with respect to the germline is recorded (Figure 4A). Mutations are stratified to silent and non-silent (synonymous and non-synonymous, respectively). These data are provided as a file and are also displayed as boxplots (Figure 4B). ASAP additionally allow conducting this step using germline sequences provided by a user.

**Figure 5:**
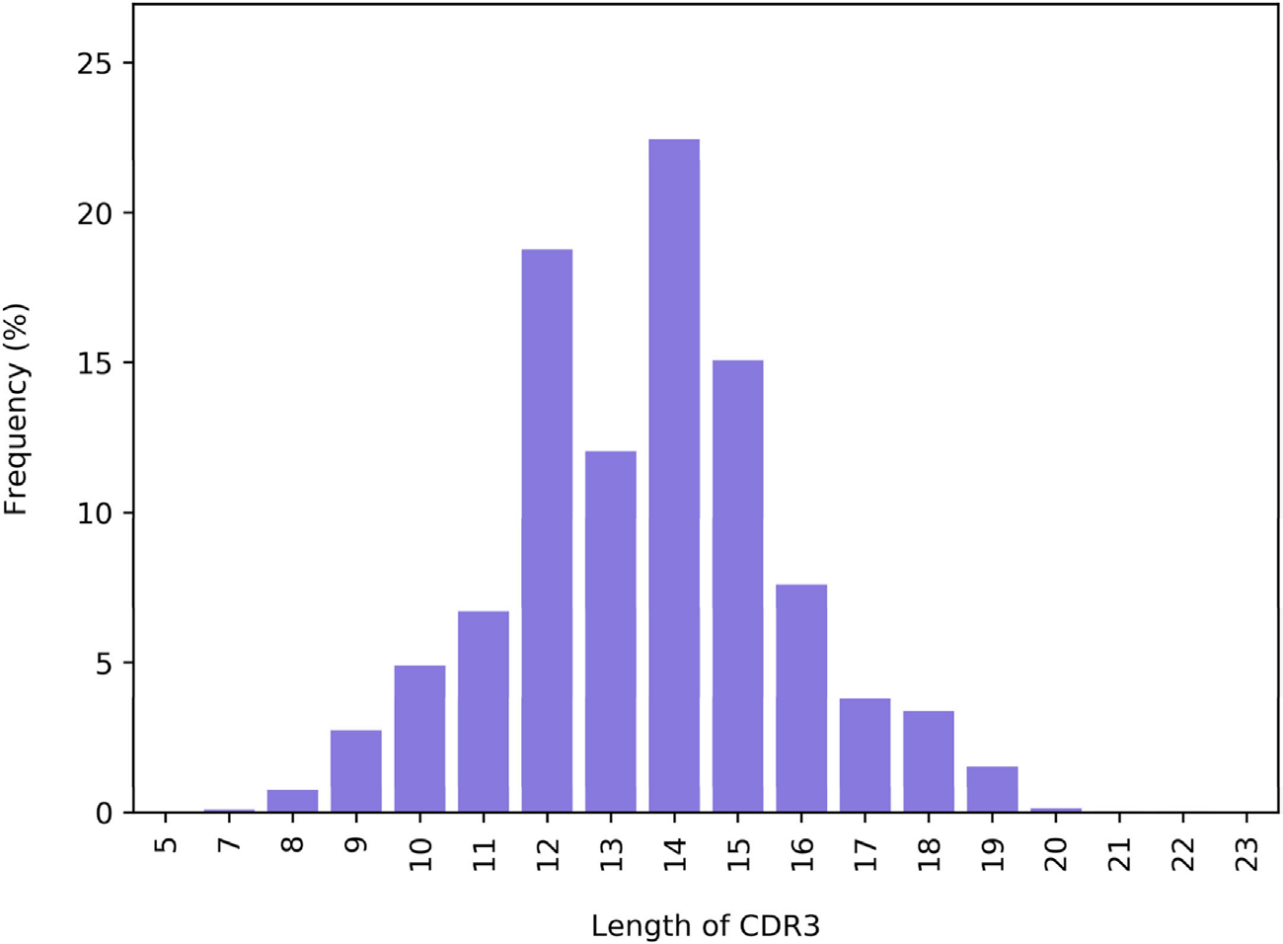
The distribution of CDR3 length (number of amino acids) in a next-generation sequencing replicate.

CDR3 length distribution was shown to vary in response to specific challenges (27–30). ASAP hence provides the distribution of CDR3 length for each replicate, both as a file and as a histogram (Figure 5).

**Figure 6:**
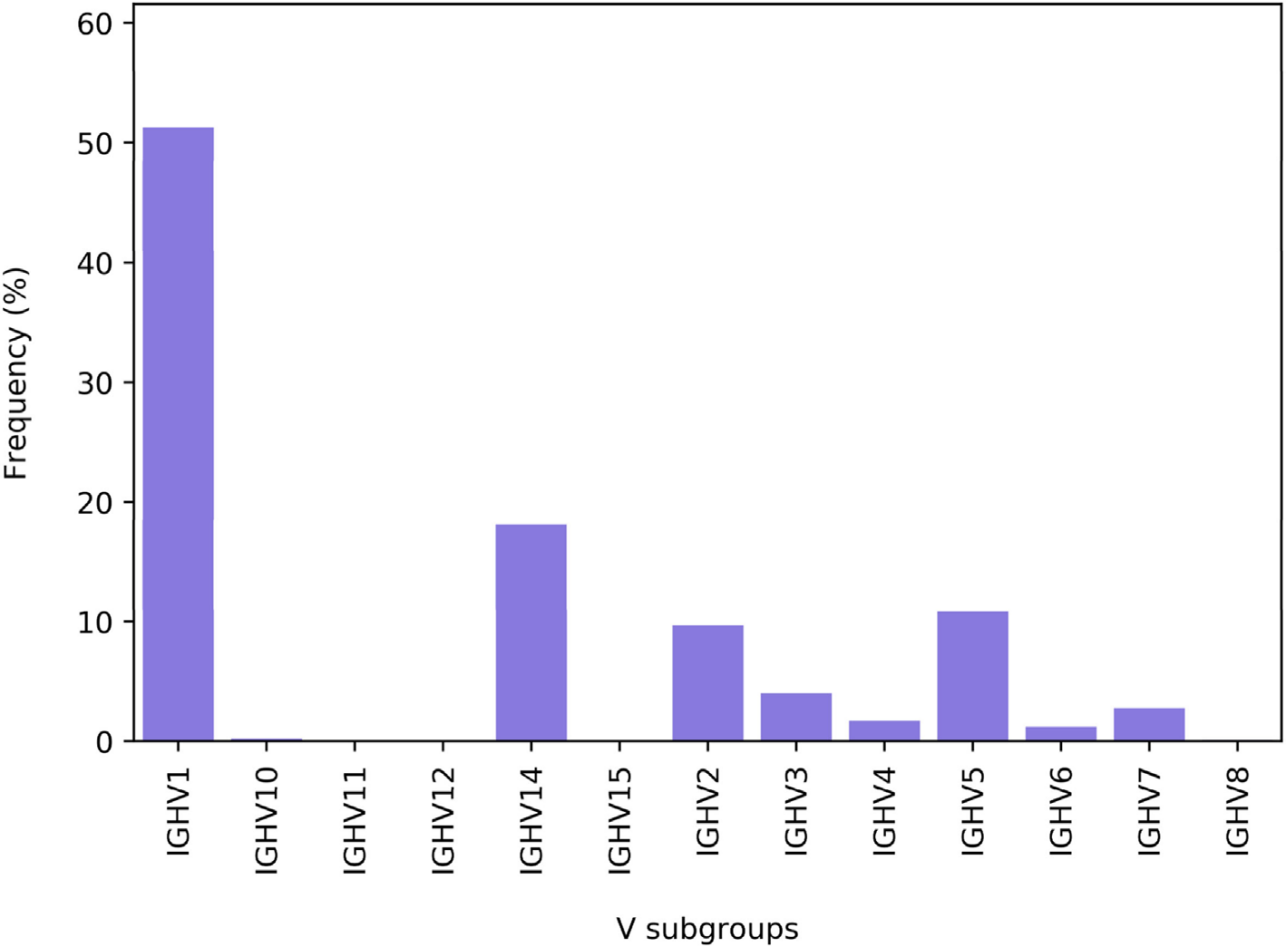
The distribution of V subgroups in a replicate. Shown is the distribution of the subgroup families for the heavy chain of IgG.

**Figure 7:**
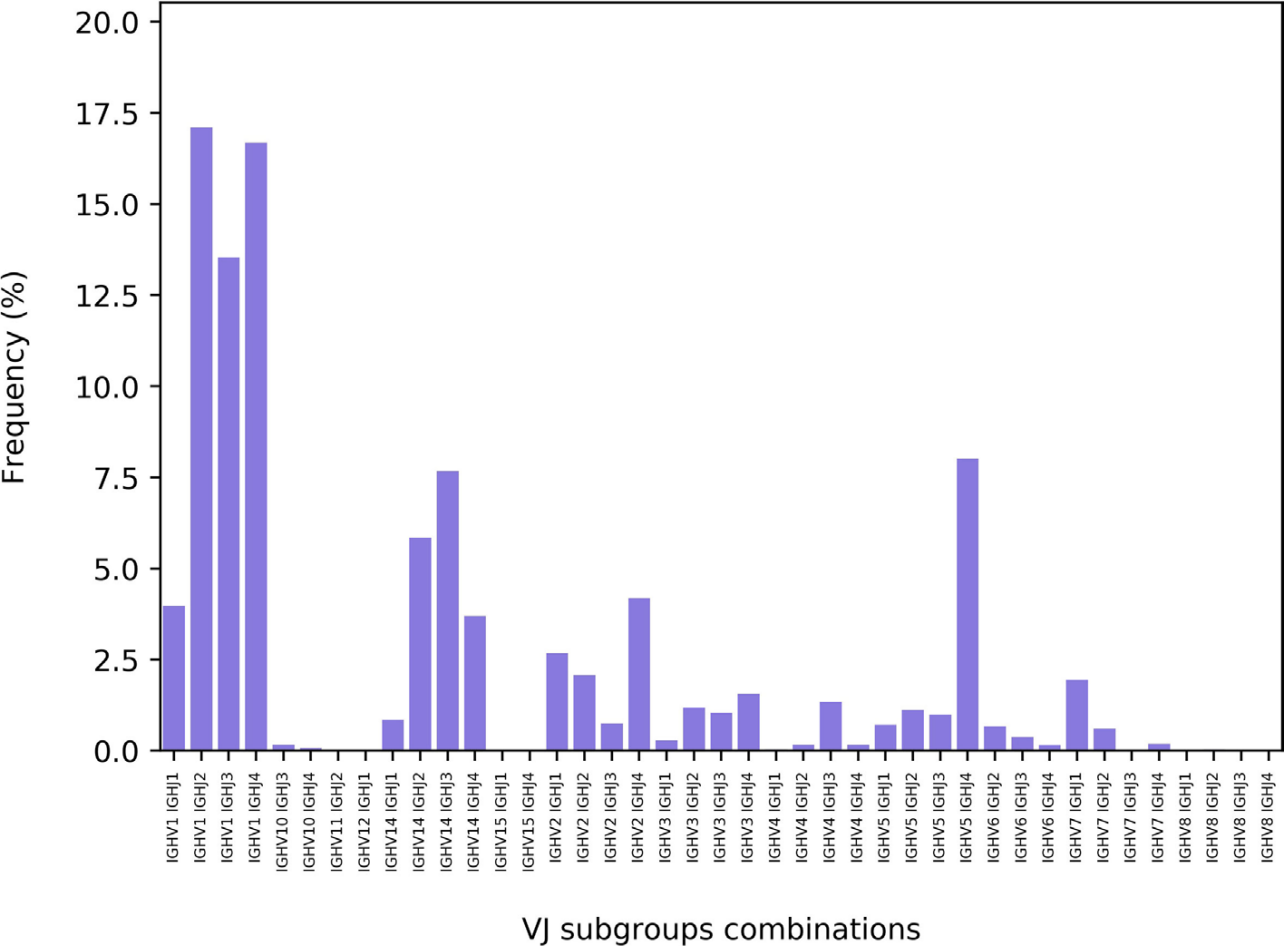
The distribution of the V(D)J combinations in a next-generation sequencing replicate. Shown are the frequencies of the various combinations between the V and J subgroups.

Each of the V, D, and J genes can be encoded by several distinct alleles termed subgroups (26). Thus, for each gene, the server provides the frequency of unique amino acid sequences included in each subgroup. These data are graphically shown as three histograms. An example of such a histogram is shown in (Figure 6). Data regarding the subgroup usage and combination were previously shown to be important for understanding the nature and dynamics of the immune response, facilitating the distinction between cell types (31, 32). ASAP thus also provides the frequencies of all possible subgroup combinations. An example of the histogram is shown in Figure 7.

**Figure 8:**
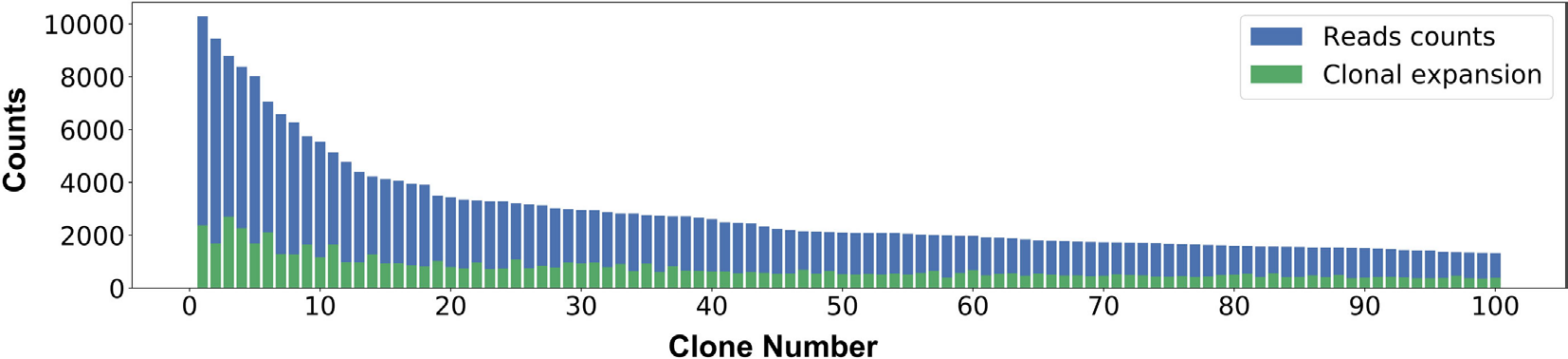
Clonal expansion. The *X* axis shows the most prevalent 100 clones. For each clone, the *Y* axis represents the number of variable region amino acid reads supporting each clone (in blue) and the number of contributing unique variable region amino acid sequences (in green).

A clone is defined as the collection of antibody sequences that likely originated from a single B cell lineage. Clonal analysis may provide insights into the evolution of the antigen-specific response of that lineage (11). In practice, clones are defined by clustering variable region sequences that comprise highly similar CDRH3 regions, although the exact definition of this similarity varies among studies (10, 15, 33). Here, we define a clone as all variable region sequences with an identical CDRH3 region (at the amino acid level). Let *y* be the number of reads that are associated with a specific clone. Some of the reads are identical, and some differ in their nucleotide sequence. Let *x* be the number of unique amino acid sequences within a clone (these sequences differ in regions other than the CDRH3 region; *x* ≤ *y*). Both *x* and *y* are biologically important: *y* is indicative of the level of sequence variance within a clone, a phenomenon called clonal expansion (2) and *x* is indicative of the proliferation tendency of the clone (or when cDNA is used, high values of *x* may also indicate high expression levels). In ASAP, the data regarding these *x* and *y* values are provided for each clone, as well as a graph showing these values for the *K* clones (the default is *K* = 100) with the highest *y* values (Figure 8).

**Figure 9:**
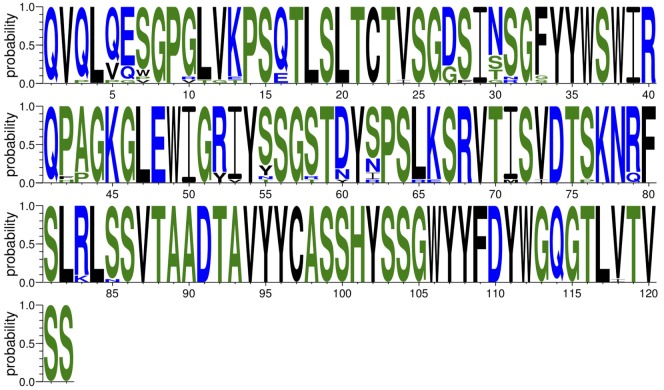
Sequence logo of one of the top clones.

For each of the above *K* clones, ASAP also provides a file for the multiple sequence alignment between all clone members. These multiple sequence alignments are also visualized by Wasabi (34). An addition annotation file includes the following information for each clone: [1] CDRH3 amino acid sequence; [2] CDRH3 counts (the *y* parameter described above); [3] unique variable region amino acid sequence counts (the *x* parameter described above); [4] the consensus sequence; [5] the amino acid sequence of the clone member which is most similar to the consensus sequence; [6] the similarity score; [7] the DNA sequence of the most similar sequence. Finally, for each clone a sequence logo graph is also provided (Figure 9).

In addition, ASAP generates a specific FASTA file for each chain type (e.g., V_H_ AA Sequences.fasta). In each such a file, all amino acid sequences of the variable region are given. For each sequence, the following information is given in its header: chain type and isotype, the CDR3 amino acid sequence, the V, D (only in V_H_), and J subgroup families, and unique variable region occurrences (at the amino acid level). For proteomic analyses, and in particular, those aimed to analyze antibody repertoires, the C terminus of the variable region sequence (i.e., the N terminal of the C_H_1 region) must include a proteolytic cleavage site (9). To this end, the server allows concatenating for each of the sequences in the above FASTA file a proteolytic cleavage site. By default, the “ASTK” and “AK” peptides are added after the FR4 motif (Figure 1B) of the heavy chain, for the human and mouse sequences, respectively. These suffixes introduce a trypsin cleavage site at the C terminus of IgG sequences. Alternatively, users can introduce other suffixes of their choice, including isotype-specific suffixes in case that non-IgG isotypes are proteomically analyzed.

ASAP provides a supportive file that maps each amino acid sequences in the abovementioned file to the associated nucleotide sequences. A file is provided for each chain type (e.g., V_H_ AA TO DNA reads, fasta). Within each file, for each amino acid sequence the following information is given: the header includes the variable region amino acid sequence itself. For each header, the nucleotide sequences that are associated with the amino acid sequences are provided coupled with the original index from the FASTQ file.

### Joint Analysis

After each replicate is analyzed as outlined above, the server also reports statistics based on replicate integration (a “joint” analysis). Importantly, while valuable information can be obtained by analyzing individual runs, the benefit of the joint analysis is that a single graph for each attribute of the data is generated based on shared reads, e.g., the top K clones based on the ensemble of all repeats. Thus, the joint analysis is beneficial for filtering out dataset specific reads, which may be unreliable, for pointing out problematic repeats, and as a platform to get characteristics and statistical measurements from the entire data. Notably, establishing a single reliable data is of vast importance for downstream applications, such as mass spectrometry (see below).

The first step in this joint analysis is to construct a joint annotation file, in which the reads from all replicates are aggregated, and which is otherwise in the same format as the individual files for each replicate analysis (individual analysis). Based on this joint analysis, ASAP produces the entire set of statistics, as described above for the single replicates (see previous section). The differences and commonalities among the multiple runs are further characterized, as outline below.

**Figure 10:**
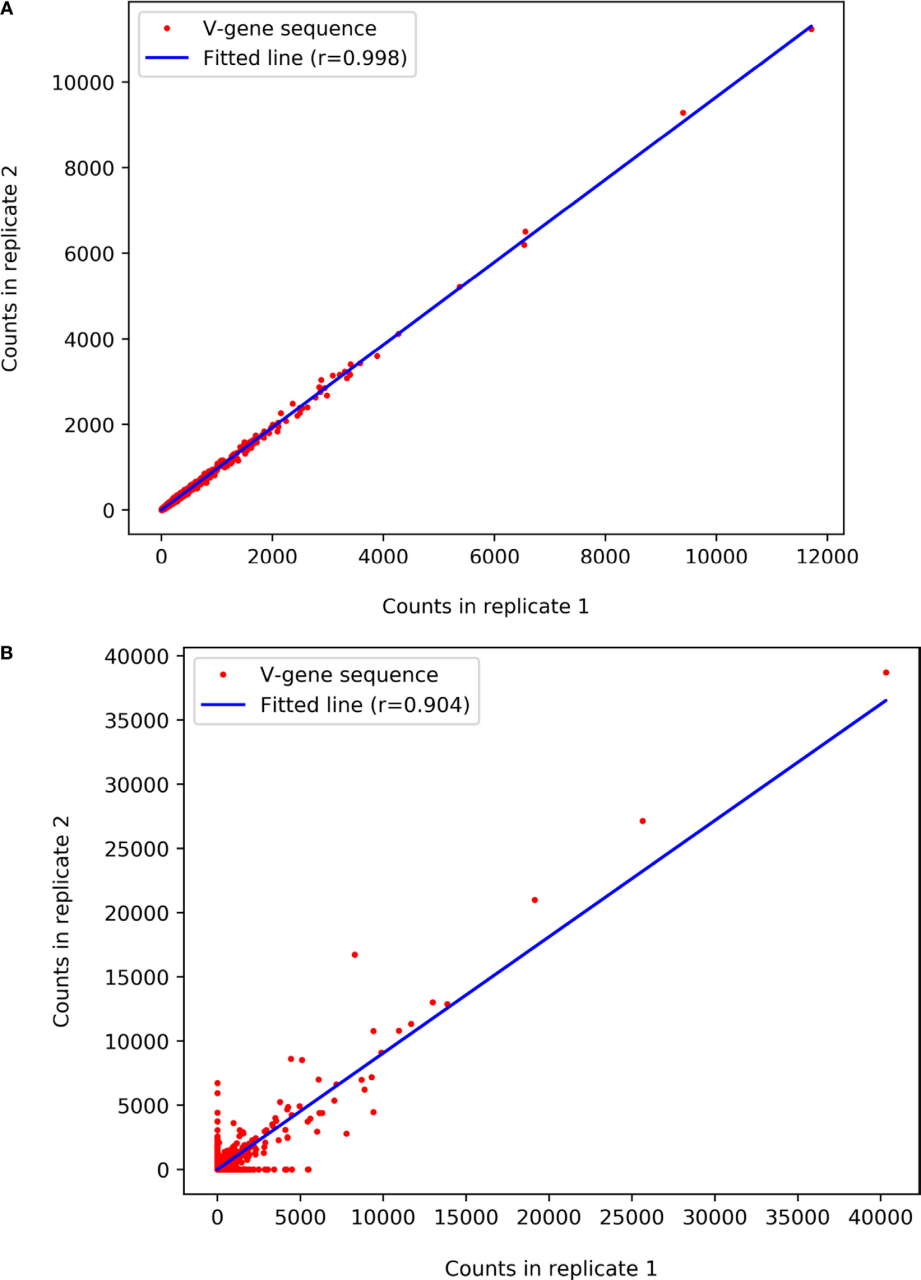
Pearson correlation between two next-generation sequencing replicates. Each dot represents a unique amino acid variable region. The *X* and *Y* axes indicate the number of times each such read appears in the first and the second replicate, respectively. **(A)** Replicates with high reproducibility and **(B)** with lower reproducibility between replicates.

The correlation between each pair of runs is reported in terms of the frequencies of each sequence. High correlation (Figure 10A) point to reproducible replicates while lower levels of correlations (Figure 10B) can point to biases that may be derived from experimental or sequencing problems.

**Figure 11:**
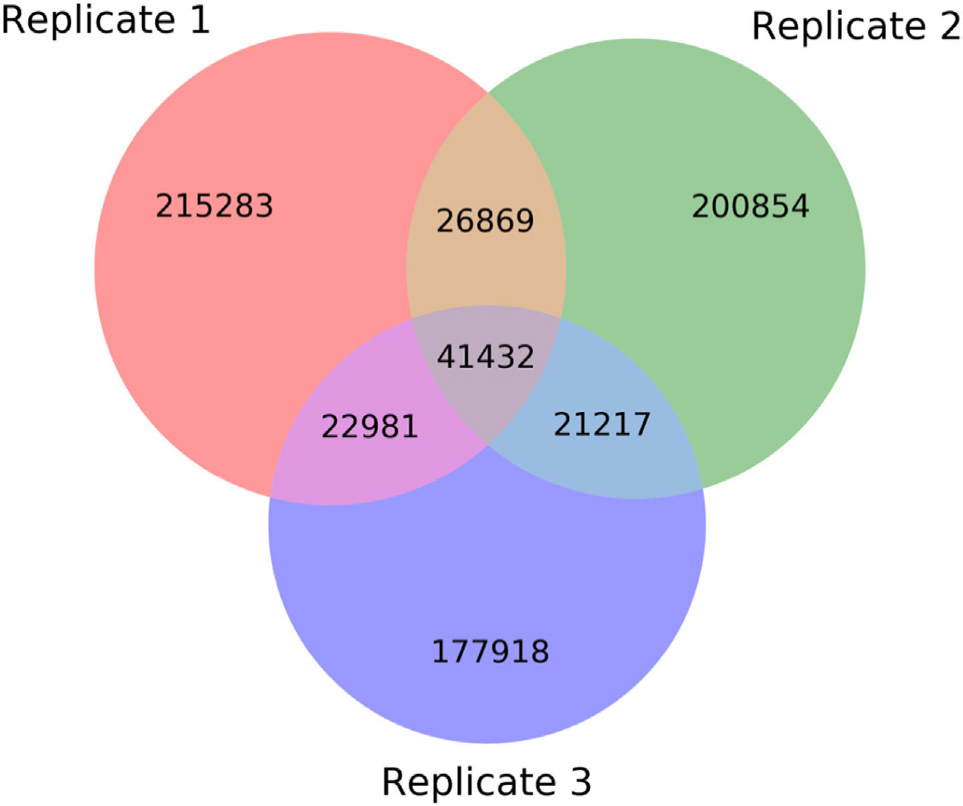
Venn diagram showing the number of variable region amino acid sequences that are shared among next-generation sequencing replicates.

Similar to the single individual processing, ASAP generates a FASTA file, which provides the entire list of amino acid sequences shared among all replicates. Unlike the individual processing file, information regarding the unique variable region occurrences summed over all replicates (at the amino acid level) and a comma separated list of these occurrences in each replicate is also provided. The server additionally provides a Venn diagram that depicts the intersections among the different replicates, presenting the number of unique variable region amino acid sequences shared between the replicates (Figure 11).

## Discussion

The ASAP webserver described here provides bioinformatic support for AIRR-Seq analysis. It is simple, free, and does not require a login information. Several webservers for analyzing AIRR-Seq obtained *via* NGS have been recently reported (35–39). However, the ASAP server offers a number of unique advantages, including the analysis of multiple replicates, defining custom search space to include new germlines, preparation of the data for proteomic analyses, and single push button analysis of raw data directly obtained from the NGS platform, without requiring any pre-analyses. This latter feature allows non-expert users to readily use ASAP for analyzing their data. Table 1 summarizes the analyses supported by ASAP compared to other related webservers.

Clonality is an important concept in antibody repertoire analysis. Yet, its exact definition varies among different studies and tools. For example, clonality may be defined based on either DNA or amino-acid sequences. Most commonly, computational clustering of variable region sequences into clones is based on the CDR-H3 region (2). Clearly, with the enormous increase in NGS data available for such studies, concepts such as clonality are rapidly evolving and choosing a specific criterion may result in too narrow or too wide clustering (under-clustering and over-clustering, respectively). Thus, clustering analysis such as the one provided in this webserver should be taken with a grain of salt when interpreting biological data.

We rely on the MiXCR software for the initial processing that uses germline information from IMGT. Novel germline alleles are inferred and discovered in an accelerated pace (40–42). Thus, it is clear that the set of germline sequences found in IMGT is restricted. This emphasizes the need to enable flexibility in defining the annotation search space to include new germlines. The inclusion of such alleles will directly affect the V(D)J usage profile, clonality, and level of SHM, thus eventually reflecting on the obtained biological insights. ASAP provides the option to append the germline space with provisional novel alleles. This option enables to annotate the AIRR-Seq data with these alleles and to inspect the impact of missing germline alleles on downstream analyses.

In various fields of biology, analyzing multiple repeats is a requirement, e.g., in expression analyses (43) or ChiP Seq data (44). The importance of repeats is critical in high-throughput analyses in order to remove random noise, thereby increasing the signal to noise ratio. While experimental and computational methodologies to increase this ratio do exist (19, 45, 46), these approaches often require sophisticated experimental setups, precluding their utilization by non-experts. Moreover, even when applying these experimental approaches, a further increase in signal to noise ratio can be achieved by experimental repeats. This motivated us to implement robust inference procedures for analyzing multiple repeats, e.g., the correlations between repeats, a Venn diagram showing the intersections among repeats, etc. Given the constant reduction in NGS costs, we expect repetitions in NGS experiments to become the standard procedure in the field of AIRR-Seq.

AIRR-Seq can also be used for proteomic identification of monoclonal antibodies within the polyclonal pool present in biological fluids. B cells effector function is the expression and secretion of antibodies into the blood or mucosal tissues. However, the composition of these antibodies remained elusive for many years. Proteomic identification of secreted antibodies requires the consolidation of a high confidence individual specific antibody archive in order to interpret the LC-MS/MS spectra. The utilization of proteomic analysis of antibodies from serum or secretion is emerging as a powerful tool to investigate their molecular composition, relative concentrations, temporal dynamics, and the relationship to well-studied B cells (6, 8–10, 12, 13, 15).

ASAP currently allows analyzing antibody sequences obtained from either human or mouse. While most studies involving AIRR-Seq focus on these two model organisms, in the future, antibody repertoire analyses from an extended taxonomical sampling should provide information about the differences among organisms, thereby providing insights into the evolution of the adaptive immune response.

## Author Contributions

AV-M, DY, OA, TP, and YW designed the research; OA, DY, HA, TP, and YW implemented the webserver; OA, TP, and YW wrote the paper; OA and AV-M equally contributed.

## Conflict of Interest Statement

The authors declare that the research was conducted in the absence of any commercial or financial relationships that could be construed as a potential conflict of interest. The reviewer UL declared a past co-authorship with one of the authors YW to the handling Editor.
